# Progress in combating cigarette smuggling: controlling the supply chain

**DOI:** 10.1136/tc.2008.026567

**Published:** 2008-09-10

**Authors:** L Joossens, M Raw

**Affiliations:** 1 Framework Convention Alliance (FCA), Brussels, Belgium; 2 Division of Epidemiology and Public Health, University of Nottingham, Nottingham, UK

## Abstract

**Background:**

The illicit tobacco trade results in huge losses of revenue to governments, estimated at $US40–50 billion in 2006, and in increased consumption and thus health problems because it makes tobacco available more cheaply. On 20 October 2008 the second meeting of the International Negotiating Body (INB2) on the illicit trade protocol of WHO’s Framework Convention on Tobacco Control (FCTC) will discuss measures to tackle the illicit trade in tobacco products.

**Methods:**

This paper presents the experience over the last decade of three countries, Italy, Spain and the United Kingdom, which shows that tobacco smuggling can be successfully tackled.

**Conclusion:**

The evidence strongly suggests that the key to controlling smuggling is controlling the supply chain, and that the supply chain is controlled to a great extent by the tobacco industry.

The illicit tobacco trade results in huge losses of revenue to governments, estimated at approximately $US40–50 billion in 2006,^1^ and in increased consumption and thus health problems, because it makes tobacco available more cheaply.^2^ In February 2008 negotiations started on a protocol to the World Health Organization Framework Convention on Tobacco Control (FCTC),^2^ to prevent illicit trade in tobacco products, and the second meeting of the International Negotiating Body on the Protocol (INB2) starts on 20 October 2008 in Geneva. Article 15 of the FCTC states that the convention should deal with all forms of illicit trade in tobacco products, including smuggling, illicit manufacturing and counterfeiting.

Evidence of the direct and indirect involvement of the tobacco industry in this large-scale fraud has become increasingly clear in recent years, on the basis of internal documents,^3^^–^6 their own admission^7^ and court judgments.^8^ In 2000 the deputy chairman of British American Tobacco, a former British minister of health, admitted: “Where any government is unwilling to act or their efforts are unsuccessful, we act, completely within the law, on the basis that our brands will be available alongside those of our competitors in the smuggled as well as the legitimate market.”^7^ In 2000 we explained that the key to smuggling in Canada “was the export by Canadian manufacturers of Canadian cigarettes to New York State (where there is no market for them as US smokers mainly smoke US brands), from where they were smuggled back into Canada. At the very least, the tobacco industry could be said to have facilitated the smuggling by supplying the cigarettes.”^9^ In July 2008 in Canada two tobacco companies pleaded guilty and admitted “aiding persons to sell or be in possession of tobacco products manufactured in Canada that were not packaged and were not stamped in conformity with the Excise Act” between 1989 and 1994.^8^ The criminal fines and civil settlements will result in the companies paying $C1.15 billion, the largest ever levied in Canada.^10^

In this paper, in which we focus on large-scale organised smuggling,^11^ we describe the cigarette export practices which targeted the illegal market in the United Kingdom, Spain and Italy, and show how substantial reductions in smuggling were achieved over the last decade.

## REDUCING CIGARETTE SMUGGLING IN THE UK, ITALY AND SPAIN

In 2002 we explained that the heart of cigarette smuggling is large-scale fraud: containers of cigarettes are exported, legally and duty unpaid, to countries where they have no market, and where they disappear into the contraband market.^12^ In this paper we describe how in the United Kingdom, Spain and Italy, over approximately the last decade, cigarette smuggling fell from around 15% to 1–2% in Spain and Italy^13^ and from around 21% to 13% in the United Kingdom.^14^ ^15^

### United Kingdom

Tobacco smuggling became a serious problem in the United Kingdom about 10 years ago. British Customs and Excise (Customs) estimated that the illicit cigarette market increased from 3% in 1996–7 to 21% in 2000–1.^14^ ^16^ In 2000–1 and 2001–2 it totalled about 16 billion cigarettes, half of them smuggled Regal and Superkings, an Imperial Tobacco brand.^16^

The nature of large-scale organised smuggling is well illustrated by these two brands. They were exported in huge quantities to places where the intended market was “unclear”, then were illegally imported through smuggling networks back into the United Kingdom. Customs believes that in 2000–1 as many as 65% of the 12 billion Regal and Superkings exported by Imperial were smuggled illegally back into the United Kingdom.^16^ From October 2000 to September 2002 a third of all Regal and Superkings were exported to the destinations in table 1.^16^

**Table 1:** Destination of Regal and Superkings exports between 2000 and 2002

Country	Exports	Population in 2002
Latvia	1363 million	2 367 000
Kalingrad (Russia)	934 million	430 000
Afghanistan	325 million	27 756 000
Moldova	576 million	4 435 000
Andorra	84 million	68 000

These export practices came under scrutiny in the UK parliament’s Public Accounts Committee hearings in May and June 2002,^16^ when members of the committee questioned the Imperial Tobacco chief executive (box 1).

**Box 1 The UK's Parliamentary Public Accounts Committee questions Imperial Tobacco's chief executive, Mike Davis**

*Committee:* … you said you believed you sold to legitimate consumers in Latvia, and in Latvia you sold 1.7 billion cigarettes in the year 1999–2000, and then the following year 1.4 billion cigarettes. Do you know the population of Latvia?

*Mr Davis:* I do not know the precise figure.

*Committee:* It is 2.3 million, which means each person, man, woman and child, including non-smokers, would have had to have smoked 722 cigarettes, which is 36 packets a year. When you were selling Regal and Superkings to this market, given it is a brand mainly sold in the UK, what did you think you were doing? Who did you think was buying these things and why did you think they were legitimate?

*Mr Davis:* I think you should understand that Latvia is a hub market, so the cigarettes were not just consumed in Latvia but in other markets in Eastern Europe. So I understand your arithmetic but the fact remains … .

*Committee:* What I find puzzling is, if it is a hub market why did it suddenly completely collapse down to 1,290,000? I said 722 cigarettes per person, that is a drop from 1999 to 2001 from 722 cigarettes per person to half a cigarette per person, that is a fairly precipitous fall in the market. What happened?

*Mr Davis:* We discontinued supply.

*Committee:* Why?

*Mr Davis:* Because product was coming back into the UK. We made efforts to identify how that was happening and we could not guarantee that we would conform to our supply policy because product was coming back, so we ceased supply.

*Committee:* So you chose Afghanistan, the source of 98% of the world’s heroin; you chose Moldova, the largest source of human prostitution in terms of women being smuggled into Western Europe; you chose Kaliningrad, which is notorious as a crime-ridden enclave of the former Soviet Union and is notorious as being run by criminal gangsters. You chose some pretty odd locations.

One comes to the conclusion that you are either crooks or you are stupid, and you do not look very stupid. How can you possibly have sold cigarettes to Latvia, Kaliningrad, Afghanistan and Moldova in the expectation that those were just going to be used by the indigenous population or exported legitimately to neighbouring countries, and not in the expectation they would be smuggled? You must know—you only have to read a newspaper every day, a member of the public could tell you—these are places which are linked to organised crime, that the drugs trade passes through all of these countries, that prostitution passes through all these countries. Did you not know that?

Source: This transcript is from the minutes of evidence of the House of Commons Public Accounts Committee, first published 2 December 2002. Parliamentary material is reproduced with the permission of the controller of HMSO on behalf of Parliament. Full transcript^16^[ is at: http://www.publications.parliament.uk/pa/cm200203/cmselect/cmpubacc/143/2061901.htm

Soon after these hearings, in March 2003, Customs reported: “In the past 18 months there has been a marked reduction in large volumes of Regal and Superkings exported to destinations outside the EU where Customs were unclear about the intended market of consumption. In Customs’ previous Memorandum to the Committee five destinations that accounted for almost a third of Imperial’s exports (around 3 billion cigarettes) were highlighted: Moldova, Latvia, Russia (including Kaliningrad), Afghanistan and Andorra. Since May 2002 exports of Superkings and Regals to these countries have reduced to only 15 million cigarettes almost exclusively to Andorra, and three of the destinations (Moldova, Afghanistan and Latvia) have not received any Imperial cigarettes at all.”^17^

**Figure 1:**
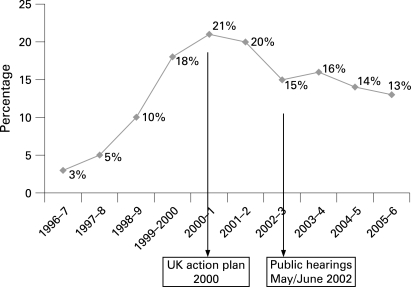
Percentage of UK cigarette market that is illegal. Source: Figure is based on data from Tackling Tobacco Smuggling (2000)^14^ and HM Revenue and Customs Departmental Autumn Performance report (2007).^15^

From 2001–2 to 2002–3 the UK illegal cigarette market share dropped from 20% to 15%. By 2005–6 the illicit market had almost halved, from 16 billion cigarettes to 8½ billion (fig 1).^18^ Regal and Superkings seizures, which represented over one billion cigarettes in 2000–1 (half of all seizures) were less than five million in 2006–7, just 1% of seizures of genuine UK brands.^15^ ^16^

In 2000 the UK government also announced an anti-smuggling action plan, which included scanners for container detection, prominent fiscal marks on packs, increased punishment, more customs officers and a campaign to increase public awareness,^14^ which halted the increase of smuggling in 2000–1 and 2001–2.

Another British government approach to smuggling has been the Memorandum of Understanding (MOU), an unenforceable, non-binding agreement, which depends for its effectiveness on goodwill.^19^ Perhaps not surprisingly the tobacco companies have been happy to sign it, Gallaher being the first in April 2002. However according to UK Customs 690 million Gallaher cigarettes were seized from 2002 to 2006,^20^ with seizures of Gallaher brands increasing since 2003–4 in spite of the MOU.^21^ The smuggling of Gallaher cigarettes into the United Kingdom is officially estimated to have cost the Treasury more than £1 billion in lost revenue since 2000.^22^ Arguably, the weakness of the MOUs is acknowledged by the UK government introducing legislation to make measures to combat illicit trade enforceable. The UK Finance Act 2006 makes it a legal duty for tobacco manufacturers not to facilitate smuggling and manufacturers who fail to take sufficient steps to prevent their products being smuggled into the United Kingdom face fines of up to £5 million.^23^

With an illegal market share of 13% in 2005–6 the problem of cigarette smuggling has not been solved in the United Kingdom, mainly because of bootlegging and the counterfeit trade. Bootlegging involves the purchase, by individuals or small groups, of tobacco products in low tax jurisdictions, in amounts that exceed customs limits, for resale in high tax jurisdictions.^11^ Bootlegging was a minor problem in the past because price differences of the same brand between countries were smaller.^24^ In 2000–1 in the United Kingdom 80% of cigarette smuggling was large-scale smuggling (container fraud) and 20% bootlegging.^25^ However, large price differences for the same brands^26^ ^27^ have grown even larger in recent years. For example in 2008 the price of a pack of Marlboro in the United Kingdom was eight times that in Russia and 12 times that in Ukraine.^28^ While large-scale smuggling with genuine brands has decreased, the proportion of counterfeit cigarettes in the United Kingdom has increased. According to UK Revenue and Customs, around a quarter of the smuggled cigarette market is now counterfeit.^29^

**Box 2 Steps in smuggling American cigarette brands into Italy**

1. The cigarettes are manufactured in the United States
2. A first purchaser places an order with manufacturer
3. The containers are exported to the ports of Antwerp in Belgium, under the “transit” regime, which allows the temporary suspension of taxes on goods destined for a third country
4. The containers are exported and imported through many different locations over a short period of time, the objective being to obscure the tracking of the goods and to make it extremely difficult to identify the real owner
5. Payments are often made in cash or from tax havens or countries with secretive banking laws, such as Liechtenstein or Switzerland
6. The containers are then transferred from the legal transit regime to the illegal domain, in a location known for its lack of surveillance, for example the warehouses in the Montenegrin ports of Zelenka, Bar and Katar
7. Cases of cigarettes are transferred to speed boats and shipped at night across the Adriatic to Italy, about 100 miles away
8. The cigarettes are sold in the streets of Naples and Bari, often by immigrants

*Sources:* references 30, 31, 33–37.

### Italy

Italy was one of the first European countries to experience a serious cigarette smuggling problem. It was concentrated mainly in some southern provinces where, in the second half of the 1980s, criminal organisations started smuggling, in Campania (particularly Naples) and Puglia (particularly Bari and Brindisi), where they took advantage of access to the Adriatic Sea.^30^^–^32

Sales of smuggled cigarettes in Italy were estimated at 1.5 million kg in 1985, 8.4 million kg in 1992 and 17 million kg in 1998, when smuggling peaked.^30^ ^31^ The smuggling involved primarily American manufactured cigarettes, especially Marlboro,^32^^–^36 and its modus operandi is summarised in box 2.^30^ ^31^ ^33^^–^37

In 1992 the Italian authorities banned the sales of Marlboro because they believed that Phillip Morris (PMI) was complicit in their smuggling.^35^ However the ban was lifted because of insufficient evidence, and later that year the government and PMI signed an MOU intended to prevent the smuggling.^31^ This MOU was strongly criticised in a 2000 Parliamentary committee report which said that it only created “an illusion of good collaboration”.^31^

By 1998 European governments and European Community (EC) officials believed that the manufacturers were selling American cigarettes to traders who resold them into black markets set up to evade foreign taxes, and had begun investigations.^38^ In November 2000 in New York, the EC filed a civil action against Phillip Morris and RJ Reynolds, accusing the companies of “an ongoing global scheme to smuggle cigarettes, launder the proceeds of narcotics trafficking, obstruct government oversight of the tobacco industry, fix prices, bribe foreign public officials, and conduct illegal trade with terrorist groups and state sponsors of terrorism.”.^33^ In 2001 ten EU countries, led by Italy,^39^ joined the lawsuit.

In 2004 the EC and member states dropped the case against Phillip Morris in return for an enforceable and legally binding agreement (which did not constitute an admission of liability by PMI).^40^ ^41^ Under the agreement PMI agreed to pay the EC $1 billion over 12 years.^40^ PMI also had to make substantial additional payments if smuggled PMI cigarettes continued to be seized by the authorities. The agreement also required PMI to control future smuggling through a range of measures, which included controlling the distribution system and contractors supplied, and tracking and tracing measures. In order to effectively combat illicit trade in tobacco products, law enforcement authorities need to be able to monitor the movement of lawfully manufactured tobacco products as they travel through the supply chain, and re-create the route taken by lawfully manufactured tobacco products that they have seized.^42^ PMI, for instance, marks master cases (containing 10 000 cigarettes) with unique, machine scannable barcode labels before selling to a first purchaser (see box 2). Since 2004 PMI has marked 200 million master cases containing 2000 billion cigarettes with such a unique code. Currently the labels are limited to the master cases but under the agreement “PMI shall maintain an ongoing program of research and development concerning methods and technologies for improving Carton and Pack Coding technologies.” In 2008 PMI is gradually introducing the tracking of the cartons in smuggling sensitive markets, such as Russia and Ukraine. At pack level PMI is experimenting and applying unique codes on the individual packs in the German and Peruvian market (the information on the PMI tracking and tracing system was collected during a visit, organised by the European Anti Fraud Office (OLAF) on 8 July 2008 in Neufchatel, Switzerland).

**Figure 2:**
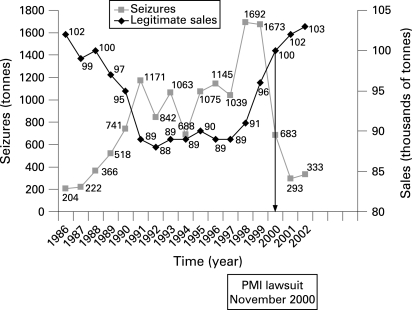
Cigarette seizures and legitimate sales in Italy 1986–2002. Source: figure is based on data from Guardia di Finanza annual reports, 1986 to 2003^43^ and Italian Institute for Statistics (2008). (Lorenzo Spizzichino, Italian Institute for Statistics (ISTAT), Italian Ministry of Health, 8 April 2008, personal communication.)

From the late 1990s onwards there was a striking fall in seizures, and legal sales—which fell from the mid 1980s to the beginning of the 1990s then were stable from 1991 to 1997 at around 89 000 tonnes per year—rose to just under 103 000 tonnes in 2002 (one tonne is about one million cigarettes) (fig 2). The volume of seized cigarettes, reflecting the amount of smuggling, was a mirror image of legal sales, decreasing from 1700 tonnes in 1998 to just 333 tonnes in 2002.^43^ Over this period American manufacturers changed their export practices such that cigarette exports from the United States to the port of Antwerp fell from 49 billion in 1997 to three billion in 2001,^44^ and cigarette smuggling fell from around 15% in the 1990s to 1–2% in 2006.^13^

Data on legal sales of foreign cigarettes in Campania and Puglia show a large increase from 1998 to 2000, reflecting how illicit foreign cigarettes became unavailable over that period, obliging smokers to buy legal foreign cigarettes. Over this period legal foreign cigarette sales increased 121% in Campania, 55% in Puglia and 19% in the whole of Italy (table 2) (Lorenzo Spizzichino, Italian Institute for Statistics (ISTAT), Italian Ministry of Health, 8 April 2008, personal communication).

**Table 2:** Legal sales of foreign cigarettes in Campania, Puglia and Italy, 1998–2000 (tonnes)

	Campania	Puglia	Italy
1998	3719	3225	59 634
2000	8231	4997	71 216

Source: Lorenzo Spizzichino, Italian Institute for Statistics (ISTAT), Italian Ministry of Health, 8 April 2008, personal communication.

### Spain

**Figure 3:**
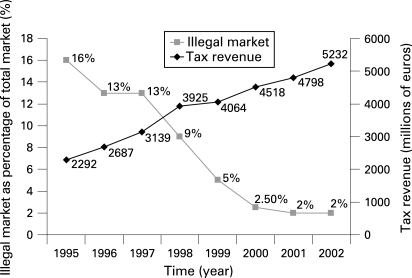
Cigarette tax revenue and the illegal cigarette market in Spain 1995–2002. Source: figure is based on data from “Evolución del contrabando de tabaco en España” (2003).^45^

From 1993–1996 to 1996–2000 the resources Spain invested in combating cigarette smuggling rose from €4 million to almost €40 million.^47^ Over this period the market share of smuggled cigarettes decreased from 16% to 2% and cigarette tax revenue rose from €2300 million to €5200 million^45^ (fig 3). An investment of €44 million led to an increase in revenue of almost €3000 million.

As with Italy, American brands (in this case mainly RJR’s Winston) were a key source for the contraband trade,^33^ so the Spanish authorities focused resources on preventing container smuggling, leading to significant seizures. For example, in 1998 Spain seized a cargo ship which had off-loaded 80 million smuggled cigarettes supplied by Reynolds. RJR Tobacco International—based in Switzerland—refused to cooperate with the investigations, claiming the protection of Swiss secrecy laws.^33^ This case resulted, for the first time, in the EU formally requesting the help of the US government in combating smuggling, based on a 1997 US-EC customs mutual assistance agreement.^38^ Evidence obtained by the European Fraud Office (OLAF) in Greece, Albania and the United States, and the verification of the origin of the markings of the seized cigarettes, led to the conviction of the smugglers in Spain in 1998.^46^

In addition, to take action against large-scale smuggling, Spain collaborated with OLAF to prevent cigarettes illegally entering the country from Gibraltar and Andorra. In Andorra this included sealing the border and political pressure on the Andorran government by the EC and member states, which forced Andorra to pass legislation making it illegal to smuggle tobacco into neighbouring countries.^47^ The Spanish customs authorities said that their success was not due to controlling distribution at street level, which is almost impossible, but to reducing supply into the country at container level, through intelligence, customs activity and improved national and European cooperation and technology.^9^ All these measures, including the investigations of US tobacco companies and the 2000 EC lawsuit they led to, resulted in the supply of American cigarettes into the illegal market in Spain being cut off.

## DISCUSSION

Anti-smuggling measures in the United Kingdom included scanners for container detection, prominent fiscal marks on packs, increased punishment, more customs officers and parliamentary hearings that exposed tobacco industry export practices. Large-scale container fraud fell significantly between 2000–1 and 2005–6. When the industry stopped exporting Regal and Superkings that were re-imported to the illicit market, there was a huge fall in customs seizures of these brands coming back into the country, dramatically illustrating that cutting off the supply to the illicit market led directly to a fall in smuggling. In Italy, following Italian and European investigations, which led to legal action against the tobacco industry, and subsequently to a binding agreement with Phillip Morris, there was a dramatic fall in customs seizures and a corresponding rise in legal sales. Supply of smuggled cigarettes into Spain was reduced by a combination of measures, including intelligence, customs activity in border areas and international cooperation, both within Europe and with US authorities over the supply of seized US brands.

The OLAF investigation of the tobacco companies in the United States in 1998 and the Spanish and Italian customs activities and ensuing lawsuit against American tobacco companies also appear to have had a significant impact. Over the period covered by these actions there was a dramatic fall in US exports to Europe. A plausible interpretation of the data is that the industry changed its export practices promptly in response to the investigations. What the investigations and threat of legal action did was change the risk-benefit equation for the industry. The prospect of a lawsuit and possible financial penalties increased the risks of supplying cigarettes that were reaching the illicit market and reduced the benefits. The changes in export practices were then reinforced and consolidated by the formal, legally binding agreement. All three examples have one common factor: smuggling was reduced by interrupting the supply chain from the manufacturers to the illicit market. In Italy and Spain international cooperation was also crucial.

There is little convincing evidence that voluntary measures, like MOUs, have a useful effect. When they are compared with the legally binding EC-Phillip Morris agreement, this does not seem surprising. For example, none of the MOUs stipulated seizure payments, whereas the EC-Phillip Morris agreement included such payments. MOUs rely entirely on the goodwill and willingness of the tobacco manufacturers to cooperate, rather than measurable outcomes. They are not legally binding, not obligatory, and if the companies fail to honour them, there are no penalties or seizure payments. When, in a recent court case involving Gallaher, the UK Customs Director General for Enforcement was asked by the judge about MOUs: “You have no lawful power to tell somebody what he may lawfully do?” the reply was: “Absolutely. We had no power.”^48^

Although we have focused in this article on large-scale organised smuggling, it is only one type of the illicit tobacco trade and a treaty to combat illicit trade needs to deal with all its forms. In the United Kingdom, for example, where the illegal cigarette market remains high, at 13% in 2005–6, bootlegging and counterfeit still result in cheap products reaching the market, thus representing a serious health problem.

These data strongly suggest that the key to controlling large-scale organised tobacco smuggling is cutting off supply to the illicit market—turning off the tap.^12^ The UK experience shows quite clearly how investigation of one tobacco company, with the implied threat of legal or punitive action, led directly to a fall in smuggling. In fact the chief executive himself explained what happened when asked why there had been a precipitous fall in one of its “unclear” export markets, Latvia: “We discontinued supply.” The data from Spain and Italy show that the industry is able to a great extent to control the supply chain, so that when it considers that smuggling becomes too risky, it stops supplying the illicit market, and its brands are no longer available in those illicit markets.

**What this paper adds**

- This paper builds on our knowledge of the nature of large-scale cigarette smuggling. We know that the tobacco industry has supplied cigarettes on a large scale that find their way into the contraband market, losing governments tax revenue, and increasing consumption and health problems by so doing.
- This paper adds data from experience in three countries over the last 10 years, which show that large-scale cigarette smuggling can be tackled, and which show that the key to tackling cigarette smuggling is to cut off supply to the illicit market. Controlling the supply chain should be at the heart of the FCTC protocol on the illicit tobacco trade.

Thus enforceable measures to control the supply chain should be at the heart of the FCTC protocol on the illicit tobacco trade.^42^ These measures should facilitate investigations into smuggling operations and make the industry liable for controlling the supply chain. They should introduce measures including licensing all participants in the tobacco business; tracking and tracing systems from the points of manufacture to all points of sale, which would help identify the point of diversion from the legal to the illicit market; traceable methods of payment; strict scrutiny procedures in the selection of contractors during the supply process, ensuring, for example, that they are all genuine companies with real addresses, employees, and do not have any criminal record; and serious financial penalties for infringements. The global scope and multifaceted nature of the illicit tobacco trade requires a coordinated international response.^2^ The illicit trade protocol is an invaluable opportunity to address the issue and should commit FCTC parties to act both domestically and internationally.^42^ ^49^
